# *OsAld-Y* on *qATS6* links to alkalinity tolerance at the seedling stage in *Oryza sativa* L. ssp. *Japonica*

**DOI:** 10.3389/fpls.2026.1716444

**Published:** 2026-03-10

**Authors:** Lei Lei, Liangzi Cao, Guohua Ding, Jinsong Zhou, Yu Luo, Lei Chen, Yang Ren, Jiangxu Wang, Kai Liu, Qingjun Lei, Yusong Miao, Tingting Xie, Guang Yang, Xueyang Wang, Wei Zheng, Shichen Sun

**Affiliations:** 1 Institute of Crop Cultivation and Tillage, Heilongjiang Academy of Agricultural Sciences, Harbin, China; 2 Northeast of National Center of Technology Innovation for Saline-Alkali Tolerant Rice, Harbin, China; 3 Heilongjiang Academy of Agricultural Sciences, Harbin, China

**Keywords:** alkalinity tolerance, fine mapping, functional verification, *japonica* rice, QTL-seq

## Abstract

Salinity and alkalinity stress is one of the main factors limiting the yield of rice. The damage to growth caused by alkaline stress is more severe than the damage caused by neutral salt stress. At present, there are limited genetic resources QTLs and genes available for rice breeders to improve alkalinity tolerance. To reveal new alkaline tolerance loci, we phenotyped 1,002 F_2:3_ lines from Teng-Xi144 (TX144, alkalinity-sensitive)×Long-Dao19 (LD19, alkalinity-tolerant) for seedling survival and ion contents under 0.15% Na_2_CO_3_. Five traits were phenotyped under 0.15% Na_2_CO_3_ to identify major QTLs for alkalinity tolerance at the seedling stage (ATS). Using QTL-seq resequencing technology and a high-density linkage map based on 4,326 SNP markers, we identified *qATS6* as a major QTL affecting seedling alkalinity tolerance, which could explain 15.33% of phenotypic variation, respectively. Within the 0.69 Mb interval, annotation, expression profile analysis, qRT-PCR and sequence analysis revealed a CDS single nucleotide polymorphism (SNP) in *LOC_Os06g40640* (*OsAld-Y*) that differentiated parental responses to alkalinity stress. *OsAld-Y* has been reported to be a functional gene related to chloroplast development. Using CRISPR-Cas9 gene editing technology, we determined that *OsAld-Y* significantly enhanced alkalinity tolerance at the seedling stage. This study identified *OsAld-Y* as an alkalinity tolerant gene, and a SNP in the CDS region of *OsAld-Y* can be used to identify transcription factors that interact with it. This provides a theoretical basis for finding the molecular mechanism of *OsAld-Y* upstream and downstream regulation of alkalinity tolerance and molecular design breeding in the future.

## Introduction

Salinity and alkalinity stress, aggravated by climate change and inappropriate irrigation, is increasingly eroding crop yields and arable land (Qadir et al., 2014). As a globally critical staple, rice is especially vulnerable, and its expansion on degraded land is threatening food security (He and Luo, 2021). Therefore, the clarification and improvement of the tolerance of rice to salinity and alkalinity is essential to develop resistant cultivars and to restore the affected agricultural land (Lei et al., 2023).

Salinity-alkalinity constrains rice throughout all stages of development by reducing the solubility of nutrients, increasing the external osmotic pressure, and disrupting the ionic balance, especially the cytoplasmic pH (Chen et al., 2009). Increased Na^+^ in soil not only disrupts the ionic energy required for metabolism but also disrupts K^+^ homeostasis, so lowering cytosolic Na^+^ is key to rice tolerance (Jini and Joseph, 2017). According to the dominant anions, these stresses separate into neutral salt (NaCl, Na_2_SO_4_) and alkaline (NaHCO_3_, Na_2_CO_3_) types, with the latter often imposing harsher effects (Tanji, 2002). Alkaline salt stress not only causes osmotic and ionic stresses, which are caused by the same neutral salt, but also its internal high-pH environment has an effect on the stability of the various physiological and biochemical processes of the plant.

Salinity and alkalinity tolerance of rice is a polygenic trait governed by a number of QTLs (Lei et al., 2020). Advances in molecular markers and high-density maps have now delivered >900 salt-tolerant QTLs and 140 stress-responsive genes across all 12 rice chromosomes, predominantly at the seedling and field stages(http://gramene.org/). While extensive QTL mapping and map-based cloning have already identified key salt tolerance genes such as *SKC1* (Ren et al., 2005) and *DST* (Huang et al., 2009), genetic dissection of alkaline stress (NaHCO_3_/Na_2_CO_3_) tolerance remains rudimentary. Fourteen alkalinity tolerance QTLs were mapped from 10 seedling-stage traits under 0.15% Na_2_CO_3_ (Cheng et al., 2008). Using 200 F_2:3_ lines under alkalinity stress, 13 QTLs for dead leaf rate (DLR) and six for dead seedling rate (DSR) were identified (Qi et al., 2008). Only a few alkaline tolerance genes have been characterized—most notably *ALT1*, cloned from a loss-of-function mutant-have been characterized, and *ALT1* mitigates oxidative damage under alkali stress yet simultaneously suppresses root elongation and tiller formation (Guo et al., 2014). Consequently, new QTLs and genes suitable for breeding rice with enhanced alkalinity tolerance remain to be discovered.

Unlike conventional QTL mapping, bulked segregant analysis (BSA) rapidly tags loci by contrasting DNA pools of extreme phenotypes (Michelmore et al., 1991). Integrating BSA with next-generation sequencing, QTL-seq further expedites candidate-gene discovery without prior markers or individual genotyping (Takagi et al., 2013). QTL-seq has now dissected loci in wheat (Dong et al., 2020), soybean (Dai et al., 2024), sorghum (Ma et al., 2022), tomato (Chen et al., 2020) and cucumber (Chen et al., 2024). QTL-seq in rice has also revealed QTLs for salt tolerance, as shown by the identification of the *OsRR22* gene from a salt-tolerant bulk selected at heading (Takagi et al., 2015). By contrasting extreme reproductive-stage yield phenotypes under salt stress in the CSR11/MI48 RIL population, two bulks were formed; a 50 k SNP chip detected 5,021 polymorphic loci within 34 QTL regions, seven of which were novel salt tolerance loci (Tiwari et al., 2016). Compared to conventional QTL mapping, the combination of QTL-seq and high-density linkage maps markedly accelerates the cloning of major-effect loci underlying rice traits (Sun et al., 2019; Kodama et al., 2018). By extracting the higher alleles from locally adapted soils, resistance genes can be directly introduced in molecular breeding without any confounding effects of affinity, allowing the rapid development of alkaline tolerant cultivars for field production (Xiao et al., 2018; Xu et al., 2020). While most reported salinity-alkalinity tolerance QTLs are anchored in IRRI (the International Rice Research Institute) varieties or southern Chinese indica/japonica germplasm, northern Chinese sources remain under-exploited. Reverse genetics has validated >300 rice salt tolerance genes, among them the transcription factors *OsbZIP20* (Baoxiang et al., 2022), *OsMYB106* (Wang et al., 2020), transporters *OsNHX2* (Fukuda et al., 2011), kinases *SIT1/SIT2* (Li et al., 2014) and *OsMAPKKK63* (Na et al., 2019). Yet transgenics often incur growth penalties and lack elite alleles, hampering breeding deployment. Marker-assisted selection, exemplified by *Saltol*, shortens breeding by 4-7 years, and only *Saltol* had been widely deployed in salt tolerance breeding (Bimpong et al., 2016; Vinod et al., 2013). Compared to related reports on the function of salt tolerance genes in rice, research on alkali tolerance related genes in rice is reported on a rare basis. The results of Liu et al., 2007 showed that *NADP-ME2* does indeed have the function of increasing the alkaline tolerance of rice under alkaline conditions. An alkali-tolerant gene has been identified by GWAS and high-density genetic linkage map (Li et al., 2020).

Under alkaline stress, the rebalance of carbon-nitrogen metabolism may be the core hub for rice to achieve ion homeostasis and osmotic protection. We hypothesized that the high pH environment first triggers the redistribution of carbon flow from photosynthetic carbon assimilation to the pentose phosphate pathway (PPP), and the resulting NADPH on the one hand provides proton driving force for the plasma membrane H^+^-ATPase and Na^+^/H^+^ reverse transporters, reducing the net influx of Na^+^ in the rhizosphere. On the other hand, by maintaining the glutathione-ascorbic acid cycle, it alleviates alkali-induced oxidative stress and protects the activity of K^+^ channel protein. At the same time, the PPP intermediate ribulose-5-phosphate can accelerate the synthesis of nucleic acid and proline, form a carbon-saving osmotic protection mechanism, and reduce the loss of K^+^ substitution. Therefore, carbon metabolism reprogramming becomes the decisive metabolic switch of alkali tolerance in rice seedling stage through the three-level linkage of reducing power supply-active oxygen scavenging-osmotic solute substitution.

## Materials and methods

### Plant materials

For QTL-sequencing and linkage mapping, alkalinity-sensitive TX144 (female parent) was crossed with alkalinity-tolerant LD19 (male parent) to develop a 1002 F_2:3_ population via single seed descent. All plants were cultivated at the Heilongjiang Academy of Agricultural Sciences’ experimental station.

### Selection of alkalinity-sensitive/alkalinity-tolerant individuals at the seedling stage

In order to create two DNA bulks for sequencing, extreme individuals were selected from a 1002 individuals F_2:3_ mapping population. Seeds were dried at 40°C for 2 days to break dormancy. Then, full seeds per material were divided into control and alkalinity groups, surface-sterilized with 2% NaClO_3_ for 10 min, washed, and soaked at 30 °C for germination. After germination, the climate chamber was set at 28°C for 12 hours during the day and 25°C for 12 hours at night, with a relative humidity of 60%. The light intensity was set at 280 µmol m^-2^s^-1^ photosynthetically active radiation (PAR). The germinated seeds of each variety were sown in pots. Before sowing, low nitrogen and medium phosphorus were mixed into the pot soil at one time (N = 50 mg, P_2_O_5_ = 100 mg) without additional topdressing. For alkalinity treatment, seedlings at the two leaf stage were treated with 0.15% Na_2_CO_3_ for 7 days, then transferred to nutrient solution for another 5 days. The range of PH value is 10.5-10.9. Next, survival days of seedlings (SDS) were recorded from seeding to death. For physiological traits observation, a second experiment was conducted following the same procedure as above. After 7 days alkalinity treatment, shoots were harvested, dried at 100 °C for 30 min and 60 °C for 1 week. Then, 0.1 g of dried sample was ground and digested with 0.1 N nitric acid at 70 °C for 8 h (Campbell et al., 2017). The shoot Na^+^ concentration (SNC), the shoot K^+^ concentration (SKC), the root Na^+^ concentration (RNC) and the root K^+^ concentration (RKC) were measured with a flame photometer (Sherwood 410) to assess seedling alkalinity tolerance. The experiment was repeated three times.

### Statistical analysis

Alkalinity tolerance data for parents and F_2:3_ lines were analyzed in SigmaPlot 12.5 and were presented as mean ± SD. Figures were produced with Origin 2018 or SPSS 18.0.

### QTL-seq analysis

Genomic DNA was extracted from leaves of parental plants and extreme individuals in two pools using the CTAB method with minor modifications (Pahlich and Gerlitz 1980). The quality of the DNA was assessed using a NanoPhotometer^®^ spectrophotometer, with a required concentration >50 ng/µl. To prepare paired-end libraries with a 500 bp insert size using the Illumina paired-end DNA sample prep kit, at least 3 g of genomic DNA from bulk pools was used. The libraries were sequenced on a HiSeq X10 NGS platform by Gene Denovo (Guangzhou, China). For high-confidence variant calling, raw reads were filtered by removing those with >10% unidentified nucleotides, >50% bases with Phred quality scores <20, and reads not aligning to barcodes. Variants were called with UnifiedGenotyper (v.3.5) (https://gatk.broadinstitute.org/hc/en-us/community/posts/360073637011-UnifiedGenotyper-in-GATK4). Clean reads from parents and two DNA pools were aligned to the Nipponbare reference genome by BWA (Li and Durbin, 2009). SNPs were called by aligning T-pool and S-pool reads to LD19 and TX144 sequences using SAMtools. PCR duplicates were removed by Picard’s MarkDuplicates tool, and the GATK was used to check the quality and filter the SNP to ensure the accuracy of the SNP. The SNP-index method was applied to calculate genotype frequency differences between pools. The ΔSNP-index was obtained by subtracting the T-pool SNP-index from the S-pool SNP-index (Takagi et al., 2013). To reduce random fluctuations due to a single mutation site, two sliding window and loess fitting methods were used to reduce the noise of ΔSNP-index value. The candidate region was determined as the common area identified by both the index-slid and index-loess algorithms. ANNOVAR was used to annotate mutations for genes, functions, and genomic regions (Wang et al., 2010). The raw Illumina sequencing data were submitted to the NCBI SRA database under accession numbers PRJNA912220, SRR22671407 and SRR22671408.

### Fine mapping for alkalinity tolerance

Fresh leaves (3 to 5 cm) were taken, crushed and frozen in liquid nitrogen. High quality DNA samples (≥10 kb fragments, 10-50 ng/mL concentration) were selected for SNP analysis based on agarose gel electrophoresis. Polymorphism in 1002 F_2:3_ lines and parents was detected using a 10 K rice GBTS chip (GenioBits technology). SNPs were screened by parental genotypes, redundant markers were removed using IciMapping 4.2’ s “BIN” module, and BINs were mapped by JoinMap 4.0 (Van Oijen, 2006). QTL analysis was performed using the mean values of three replicates for each trait, with inclusive composite interval mapping in IciMapping 4.2. The LOD threshold has been set at greater than 2.5, with a 1 cM walking speed.

### RNA extraction and quantitative real-time PCR analysis

At the two-leaf stage, control plants were grown normally, while stress-exposed plants received 0.15% Na_2_CO_3_. Leaf samples from TX144 and LD19 were collected at 0 and 12 h, then frozen at liquid nitrogen. Total RNA (2 g) was extracted with TRIzol reagent, and cDNA was synthesized using Invitrogen’ s Superscript II Reverse Transcriptase kit. Quantitative real-time PCR (qRT-PCR) was performed on a Roche Light Cycler 2.10 system with 2Fast qPCR Master Mix. Gene-specific primers have been designed with Primer 5.0 software (**Supplementary Table 1**). Measurements included three biological and three technical replicates. *Actin1* (*LOC_Os03g08010*) served as the internal control. The relative expression of the genes has been calculated using the 2^-ΔΔCt^ method (Livak and Schmittgen, 2001).

### Candidate gene sequencing and sequence alignment

Candidate genes were PCR-amplified from LD19 and TX144, sequenced, and aligned to the Nipponbare reference with DNAMAN.

### Generation of transgenic rice plants

For *OsAld-Y* knockdown plants, plasmids were delivered into *Agrobacterium tumefaciens* EHA105 and transformed as in Li et al., 2017. Two PAM-containing targets (5’-GGAGAGGTACTGGAACGCGCCGG-3’ and 5’-GCCCAACCGCCAGGCGCTCCGGG-3’) were selected. 300-500 bp amplicons from edited lines were PCR-amplified, Sanger-sequenced, and decoded. Through the off target function in CRISPR-GE, potential off-target sequences were excluded (Ma et al., 2015). Edited plants were confirmed with primers *OsAld-Y-*F: CCTTGTCCACCTTGATCCCC and *OsAld-Y-*R: CCAGCATCAACGTGGAGAAC.

## Results

### Alkalinity tolerance screening and assessment at the seedling stage

**Figure 1:**
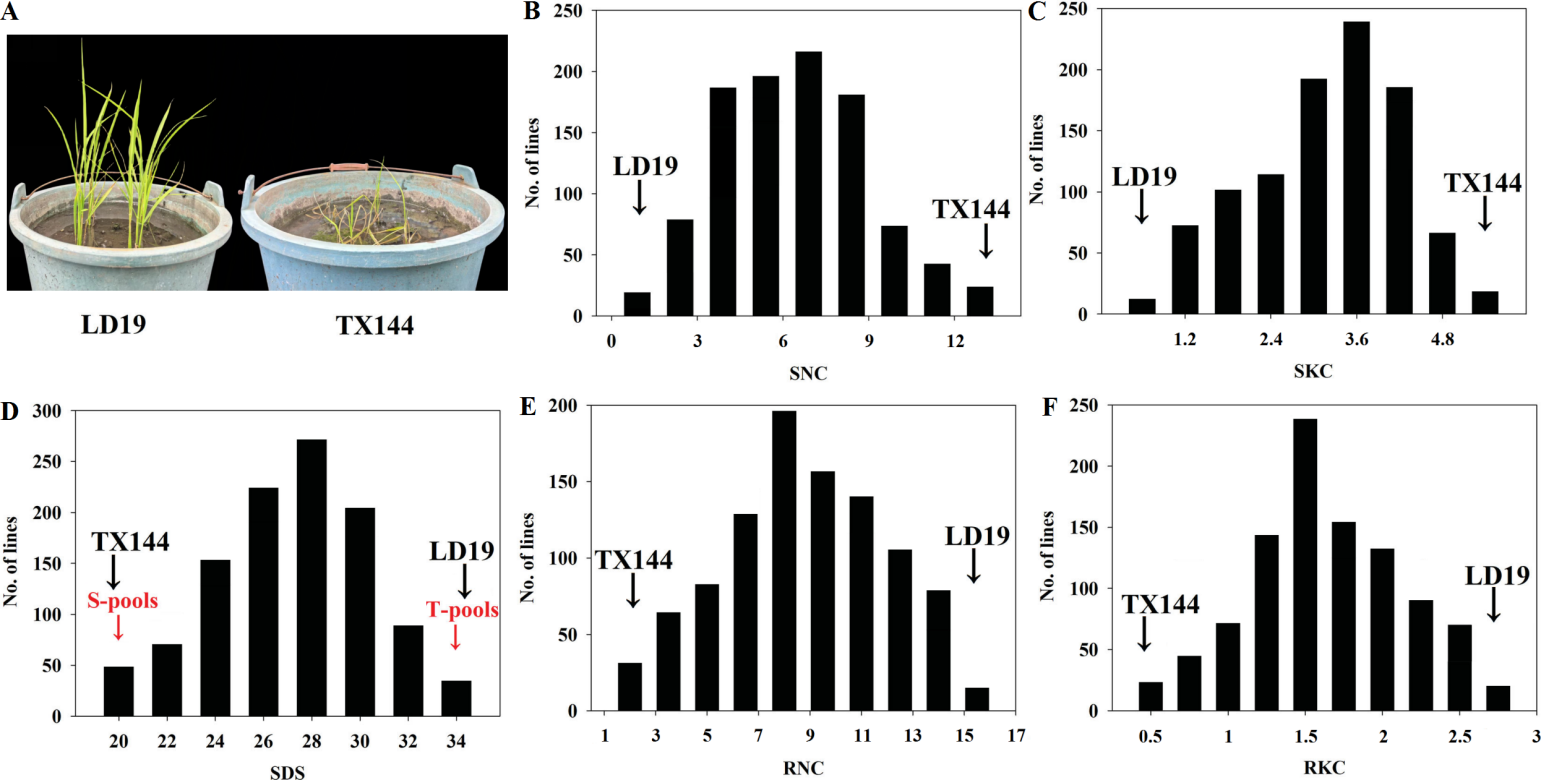
Seedling-stage performance of parents and progeny under alkalinity stress. **(A)** Growth comparison of the two parental lines. **(B)** Frequency distributions for the shoot Na^+^ concentration (SNC). **(C)** the shoot K^+^ concentration (SKC). **(D)** Frequency distributions for survival days of seedlings (SDS). **(E)** the root Na^+^ concentration (RNC). **(F)** the root K^+^ concentration (RKC).

Phenotyping revealed TX144 as alkalinity-sensitive and LD19 as tolerant, confirming LD19’ s superior alkalinity resistance (**Figure 1A**). An F_2:3_ population of 1002 lines from TX144×LD19 was screened for SDS, SNC, SKC, RNC and RKC at the seedling stage under alkalinity and control conditions. Of the 1002 lines (SDS 19-34 d), the 50 most and 50 least tolerant were pooled as T-pool and S-pool for QTL-seq (**Figure 1B**). SDS in the S-pool was markedly reduced relative to the T-pool, suggesting that the alkaline stress had a more pronounced phenotypic effect on the susceptible group. All five traits showed a continuous, approximately normal distribution, which is the hallmark of quantitatively inherited characteristics (**Figures 1C–F**). Their absolute skewness and kurtosis values remained below 1, confirming distributional symmetry and mesokurtosis, thereby satisfying the statistical prerequisites for reliable linkage mapping analysis (**Supplementary Table 2**).

### Correlation analysis

Pearson analysis revealed strong inter-trait links under alkalinity (**Supplementary Table 3**). SDS correlated negatively only with SNC, whereas SNC and SKC showed a negative correlation. There was a significant positive correlation between RNC and RKC, indicating that both were affected by salinity. The results showed that the five traits were greatly affected by the phenotypic value after alkalinity stress.

### QTL-seq analysis

A total of 428,941,568 bp of clean reads was generated from both parent pools and two F_2_ pools, with all Q30 scores reaching 85%. The parents and two pools had an average sequencing depth of 50×. Using the Nipponbare reference genome, 1,045,832 SNPs and 174,538 indels were initially identified. After trimming and filtering, the dataset was reduced to 428,791 SNPs and 70,971 indels. Finally, 499,762 high quality SNPs and indels were selected for QTL-sequencing analysis. An association analysis was performed with the SNPs identified between the T-pool and S-pool (**Supplementary Table 4**).

**Figure 2:**
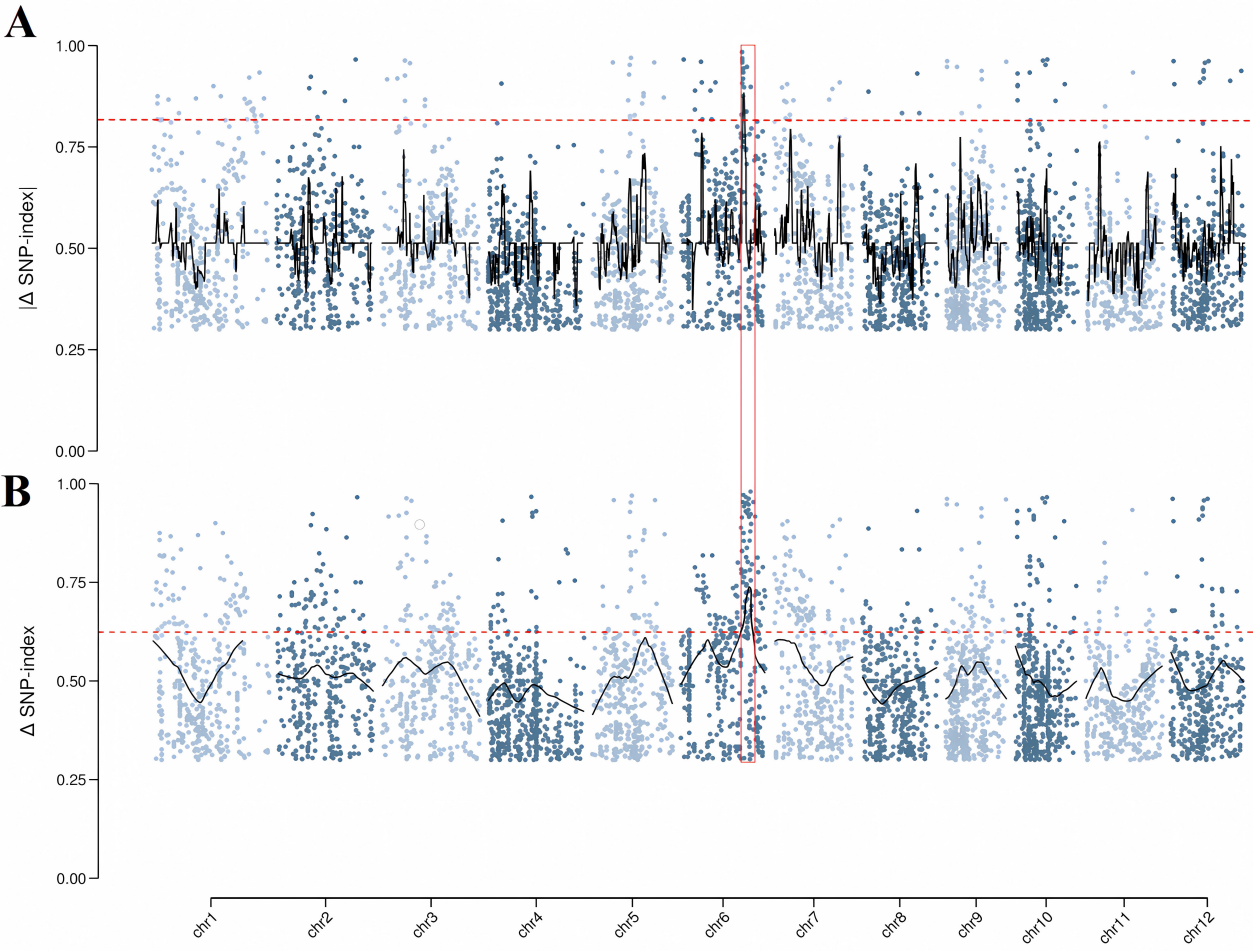
QTL-seq mapping of rice alkalinity tolerance at the seedling stage. **(A, B)** Manhattan plots for index-slid and index-loess across chromosomes. The red line represented the use of 0.995 quantiles as a threshold for sliding window results. Chromosome numbers were indicated on the x-axis.

We employed two algorithms to identify QTLs associated with alkalinity tolerance at the seedling stage. As shown in **Figure 2** and **Supplementary Table 5**, using the index-slid method, the region was detected on chromosome 6 (0.67 Mb). The index-loess method identified a region of chromosome 6, spanning 3.41 Mb. The interval identified by the index-slide method was completely covered by the interval identified by the index-loess method, so finally we selected intervals (23.13-26.54 Mb), named *qSDS6*, that contained more candidate genes for precise localization.

### Linkage mapping analysis

A 10 K SNP array identified 4,326 SNPs across 1002 F_2:3_ lines and parents; these were condensed into 1,107 bin markers (IciMapping 4.2) spanning 2,252.63 cM on 12 chromosomes with a mean interval of 2.03 cM (**Supplementary Figure 1**).

In the linkage mapping analysis, 14 QTLs were identified on chromosomes 1, 3, 4, 6 and 9. The contribution rate ranged from 4.60%-17.68%. Among them, two QTLs, *qSNC1* and *qSNC6*, were identified to be associated with shoot Na^+^ concentration. The phenotypic variation values explained by *qSNC1* and *qSNC6* were 9.29% and 17.68%, respectively, and the additive effect values were 0.80 and -1.19, respectively. Four QTLs (*qSKC1-1*, *qSKC1-2*, *qSKC6* and *qSKC9*) related to shoot K^+^ concentration were identified, which were located on chromosome 1, 6 and 9, respectively, and the PVE were 16.79%, 7.85%, 15.01% and 4.6%, respectively. A QTL, *qRNC6*, associated with root Na^+^ concentration was identified on chromosome 6, explaining 13.12% of the phenotypic variation. Four QTLs (*qRKC1*, *qRKC4*, *qRKC6* and *qRKC9*) related to root K^+^ concentration were detected on chromosomes 1, 4, 6 and 9 respectively. Three QTLs (*qSDS3*, *qSDS6-1* and *qSDS6-2*) related to survival days of seedlings were detected on chromosomes 3 and 6, of which qRCL11 had the largest PVE of 14.24%, and the LOD peak (7.04) was located at 107 cM on chromosome 6. Among them, *qSNC6* had the highest PVE of 17.68%, and the LOD peak (8.97) was located at 105 cM on chromosome 6 (**Supplementary Table 6**).

**Figure 3:**
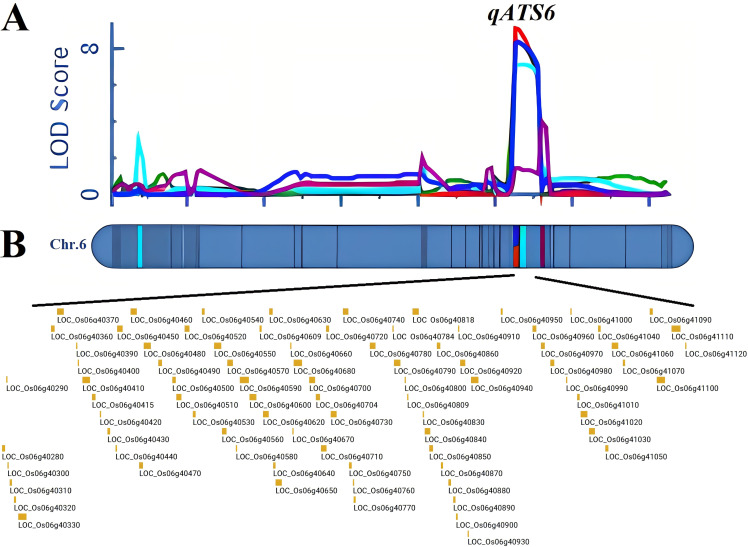
Fine mapping of *qATS6*. **(A)** Regional localization was achieved with the ICIM module of QTL IciMapping 4.2 using the high-density linkage map. **(B)** Candidate alkalinity tolerance genes within the *qATS6* interval.

It was noteworthy that *qRKC6* controlling root K^+^ concentration, *qSNC6* controlling shoot Na^+^ concentration, *qSKC6* controlling shoot K^+^ concentration and *qSDS6-2* controlling survival days of seedlings were all located in the same QTL interval, and *qRNC6* was located near the interval of the four QTLs (**Figure 3A**). The PVE of *qRKC6*, *qSNC6*, *qSKC6*, *qRNC6* and *qSDS6-2* were all greater than 10%, which were the major QTL for alkalinity tolerance at the seedling stage, and the synergistic alleles of the above five QTLs were derived from LD19. These five QTLs were uniformly named *qATS6*, and the left marker and right marker of the co-location region was 23966049 and 24660723, respectively. Within this 0.69 Mb region, 74 genes were predicted using the RAP-DB database (http://rapdb.dna.affrc.go.jp/) (**Figure 3B**, **Supplementary Table 7**).

### Candidate gene analysis

**Figure 4:**
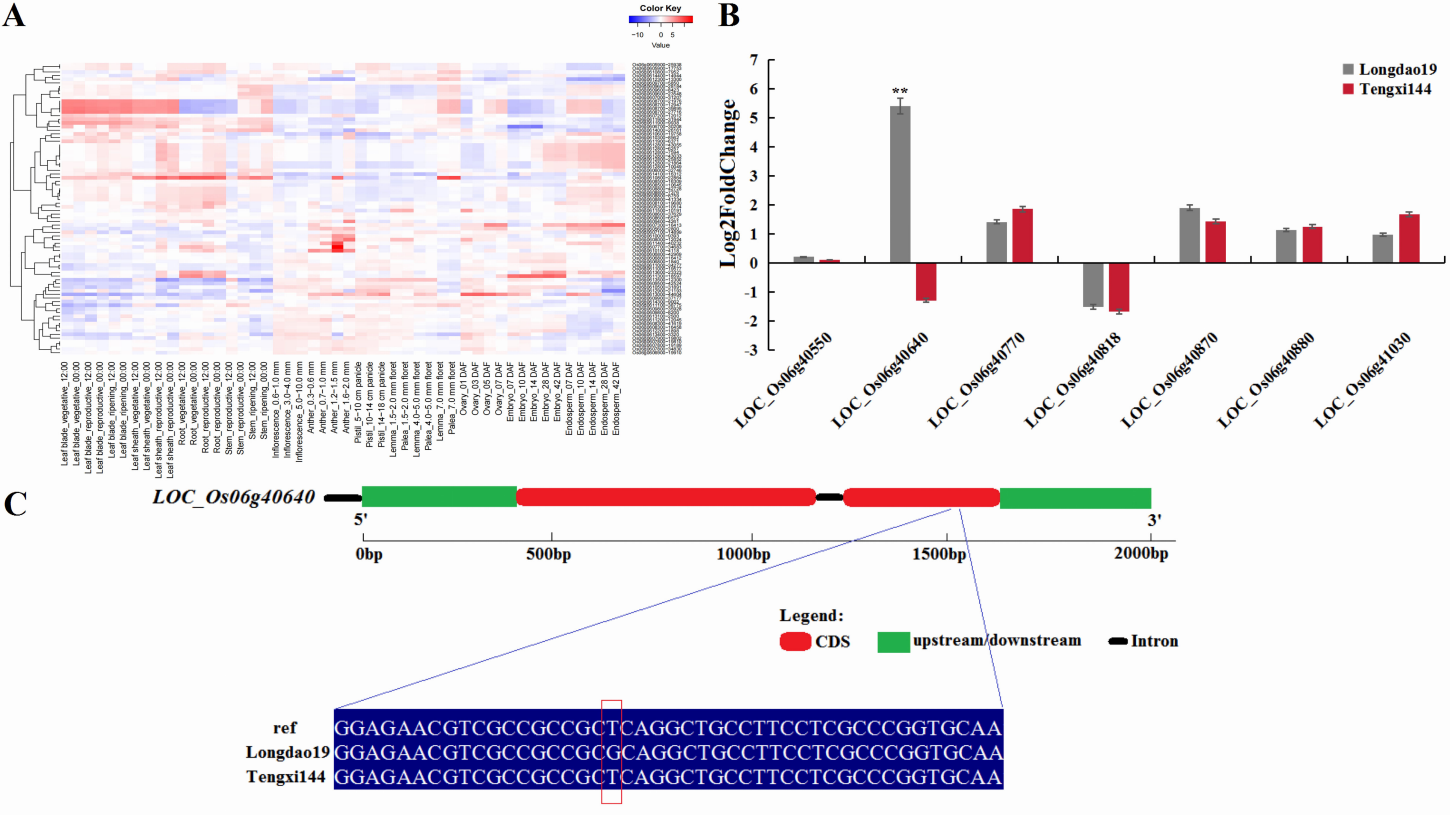
**(A)** The heat map of 74 candidate genes calculated by the rice expression profile database. **(B)** The expression level of selected genes in LD19 and TX144 12h after exposure to alkalinity stress. Log2-fold change was calculated for gene expression under alkalinity stress compared with control. **P<0.01; Student’ s t-test. **(C)** Sequence difference analysis of *LOC_Os06g40640*. The gene structure of *LOC_Os06g40640* and sequence differences in *LOC_Os06g40640* between LD19 and TX144. Ref is the reference sequence of Nipponbare genome.

To reduce the number of candidate genes and to identify the final target genes, we used the rice expression profile database (RiceXPro, https://ricexpro.dna.affrc.go.jp/) to mine the expression profile data of 74 candidate genes in the whole growth process of rice plants under natural field conditions, rice seedlings treated with various plant hormones, and specific cell types/tissues isolated by laser microdissection. Finally, the following seven genes were found to have the highest expression levels, including *LOC_Os06g40550*, *LOC_Os06g40770*, *LOC_Os06g40870*, *LOC_Os06g40880*, *LOC_Os06g40818*, *LOC_Os06g40640* and *LOC_Os06g41030* (**Figure 4A**).

To verify the expression characteristics of the seven genes under alkalinity stress, their expression patterns under alkaline stress and control (no stress) conditions were determined by qRT-PCR. The validation results for the seven genes are shown in **Figure 4B**. Under alkalinity stress, *LOC_Os06g40550*, *LOC_Os06g40770*, *LOC_Os06g40870*, *LOC_Os06g40880* and *LOC_Os06g41030* were up-regulated in both LD19 and TX144. *LOC_Os06g40818* were down-regulated in both LD19 and TX144. Only *LOC_Os06g40640* was significantly up-regulated in LD19 and down-regulated in TX144 under alkalinity stress, suggesting that *LOC_Os06g40640* is involved in the regulation of alkalinity tolerance in rice.

In order to further provide robust evidence for the identification of the most possible candidate gene, *LOC_Os06g40640* was sequenced in LD19 and TX144. Sequence analysis showed no difference in the promoter region of *LOC_Os06g40640* between LD19 and TX144, and *LOC_Os06g40640* showed that in the CDS region, one SNP (T to G, 24228830 bp) was detected in LD19 compared with TX144 (**Figure 4C**). The SNP was a synonymous mutation, but even if the SNP was a synonymous mutation, it could still fine-tune the protein expression level by changing the stability of the mRNA secondary structure or translation efficiency.

Therefore, *LOC_Os06g40640* was considered to be the most possible functional gene underlying *qATS6*. The candidate gene *LOC_Os06g40640* was previously studied and named as *OsAld-Y* in a prior study (Zhang et al., 2016). This gene was involved in chlorophyll accumulation, chloroplast development and plant growth by affecting photosynthetic rate and sugar metabolism in rice leaves.

### Characterization of alkalinity tolerance of *LOC_Os06g40640* using knockout plants

**Figure 5:**
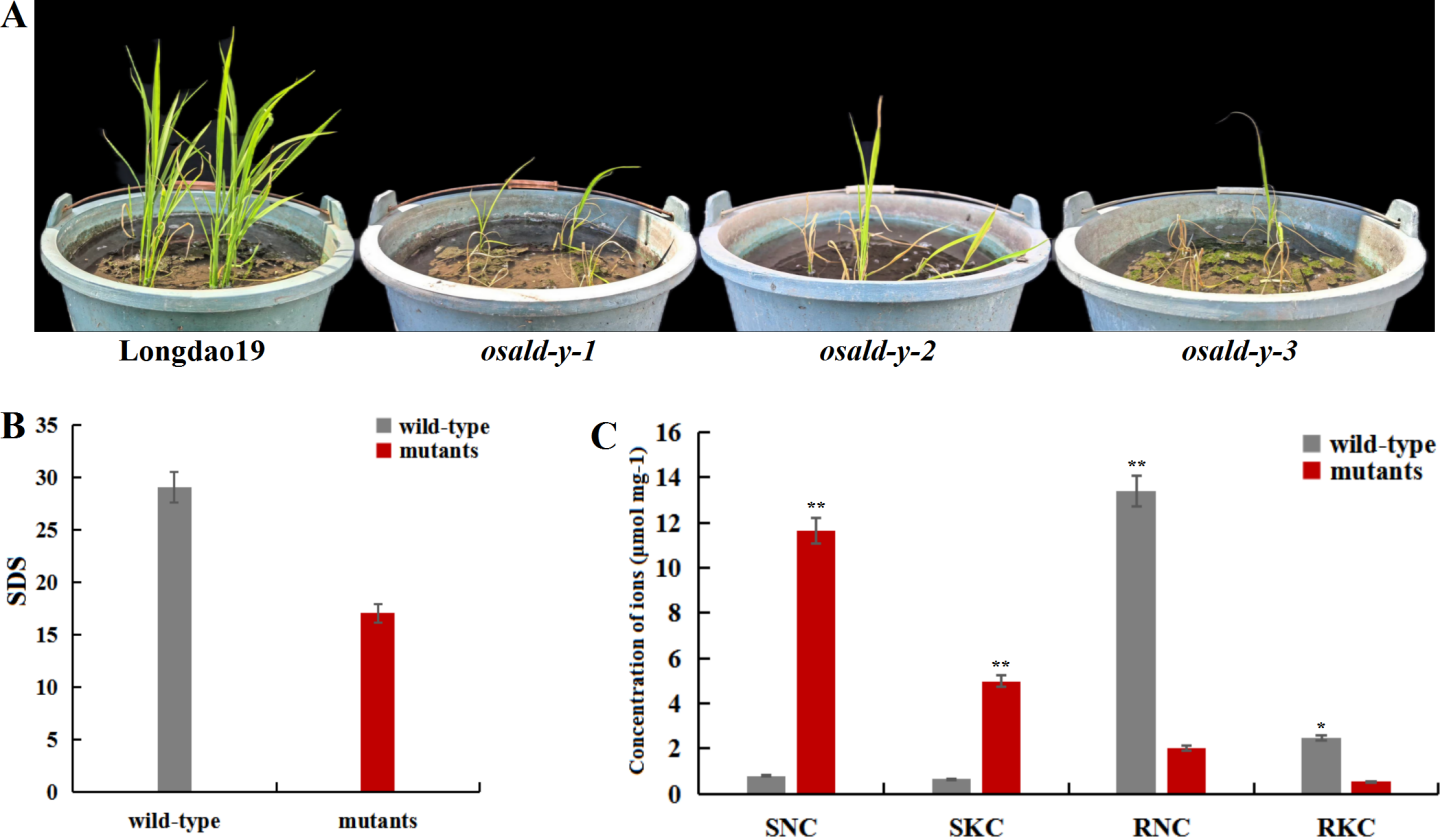
Functional characterization of the fructose-1,6-bisphosphate aldolase *LOC_Os06g40640* (*OsAld-Y*). **(A)** Alkalinity tolerance phenotype of *OsAld-Y* knockout (KO) and wild-type (WT) plants after 10 d of alkalinity stress followed by 12 d of recovery in non-alkalinity medium. **(B)** Seedling survival days (SDS) under alkalinity stress in KO versus WT. **(C)** Na^+^ and K^+^ concentrations in shoots and roots of KO and WT after alkalinity stress. **P < 0.01, *P < 0.05, Student’ s t-test.

Next, we verified the alkalinity tolerance of *LOC_Os06g40640* (*OsAld-Y*) using knockout lines generated by clustered regularly interspaced short palindromic repeats (CRISPR)/CRISPR-associated nuclease 9 (Cas9)-mediated gene editing. After the T_1_ positive plants were self-crossed to the T_2_ generation, two homozygous mutant plants were identified by first-generation sequencing, named CR-3 and CR-5. Compared with the wild-type strain, the CR-3 strain contained an A-base insertion in the sequence of the CDS region, and the CR-5 strain contained a T-base insertion in the sequence of the CDS region. The sequence of CR-3 was GCCCAAACCGCCAGGCGCTCCGGG, and the sequence of CR-5 was GCCCAACCGCTCAGGCGCTCCGGG. After 10 d of 0.15% Na_2_CO_3_ followed by 12 d recovery, nearly all *osald-y* seedlings died, whereas LD19 survived (**Figures 5A, B**). Ion profiling showed *osald-y* accumulated >2-fold SNC and SKC relative to LD19, while the RNC and RKC were significantly reduced (**Figure 5C**). Thus, *OsAld-Y* significantly enhances seedling-stage alkalinity tolerance.

## Discussion

Salinity and alkalinity stress is a major abiotic stress affecting rice production around the world. Unlike salt stress, alkaline stress not only causes plant ion toxicity, but also affects the normal plant growth because of its high pH which destroys the stability of the cells (Chen et al., 2009). Salt-alkaline tolerance in rice is an extremely complex trait. Multiple strategies have uncovered numerous salt tolerant genes in rice; nevertheless, only a handful have been shown to enhance tolerance under alkaline stress. Phenotypic identification is an important genetic tool for studying alkali tolerance in rice. In previous studies, different methods have been used to assess the alkali resistance of rice, among which the determination of Na^+^ and K^+^ concentrations is one of the commonly used methods (Li et al., 2019). Earlier studies have shown that alkaline tolerant plants compartmentalize Na^+^ into vacuoles at the cellular level, increasing tolerance to elevated ion concentrations (Wang et al., 2015). Alkalinity tolerance at the seedling stage was regulated by the balance of Na^+^ and K^+^ in the roots and shoots. As shown in **Figure 1**, in response to alkalinity stress, compared with the alkalinity tolerant cultivar LD19, alkalinity sensitive variety TX144 accumulated excessive Na^+^ and K^+^ in leaves. The data in **Supplementary Table 3** showed a negative correlation between SKC and SNC (r^2^=-0.59**), indicating a competition between Na^+^ and K^+^ in shoots, and there was a negative correlation between the SDS and SNC (r^2^=-0.64**). However, in roots, the relationship between Na^+^ and K^+^ was synergistic. In related traits, QTLs have often been found in the same region of the chromosome. *qATS6* linked with SDS, SNC, SKC, RNC and RKC, implying either pleiotropic effects or tight linkage of adjacent genes and underscoring its pivotal role in seedling-stage alkalinity tolerance in both parents.

Traditional map-based cloning technology is difficult to obtain high-density maps and near-isogenic lines, while QTL-seq technology can quickly locate major loci and has become an important tool for analyzing salt tolerance of crops. However, it typically flags hundreds of candidates in each interval, which still require functional validation (Wu et al., 2020; Lei et al., 2021). Another strategy for identifying candidate genes is to develop molecular markers in the detection interval and fine-map the QTL region through linkage mapping. However, because alkalinity tolerance is a complex quantitative trait of crops, it is necessary to mine differential fragments that confer alkalinity tolerance throughout the genome. Therefore, we used QTL-seq combined with linkage analysis to locate overlapping peak intervals (**Figures 2**, **3**). *qATS6* was a major QTL detected on chromosome 6, contributing to 14.39%, 17.68%, 15.01%, and 14.24% of the phenotypic variations for RKC, SNC, SKC and SDS respectively, whereas *qRNC6* was located near the interval of the four QTLs. No QTLs associated with alkaline stress were detected within the interval. Therefore, loci can be used as new alkaline-tolerant targets in LD19 for further molecular breeding, and the other 9 alkali-tolerant QTLs also laid the basis for subsequent gene mining research. Compared with the related reports on the function of salt-tolerant genes in rice, the research on alkali-tolerant genes in rice is rarely reported. In this study, both TX144 and LD19 were northern *japonica* rice. The results of QTL-seq and linkage analysis showed that the SNPs in the confidence interval caused phenotypic separation, indicating that there were alleles that could distinguish alkalinity tolerance in these two varieties, but it was not clear whether the SNP difference in the CDS region of *LOC_Os06g40640* was the cause of the difference in alkalinity stress response between TX144 and LD19. To solve this problem, we will clone *LOC_Os06g40640* alleles, generate plant and yeast expression vectors, and introduce them into rice protoplasts, yeast and *Arabidopsis* to assess how natural variants influence transcript abundance and sub-cellular localization under alkalinity stress.

Of the 74 candidate genes, 14 genes were cloned, and two genes were confirmed as related to alkaline stress. Genetic analysis showed that the mutation of *OsSOS2* resulted in the decrease of cesium content in *lcs1*, and *OsSOS2* encoded serine/threonine protein kinase and participates in the plant salt hypersensitivity pathway (Ishikawa et al., 2017). *OsPHL7* was involved in maintaining Pi homeostasis and enhancing rice tolerance to Pi deficiency and salt stress (Yang et al., 2024). The above results confirmed the accuracy of the interval positioning from the side. *LOC_Os06g40640* (*OsAld-Y*) had been previously reported. The mutant *ygdl-1* showed yellow-green leaves, significantly reduced photosynthetic rate, decreased chlorophyll and carotenoid content, and increased chlorophyll a/b ratio throughout the development stage. The chloroplast development was seriously flawed, the thylakoid membrane system was damaged, the grana was stacked in a disorderly manner and the grana membrane was missing (Zhang et al., 2016). Based on this, the study of the alkalinity tolerance mechanism of *OsAld-Y* should consider the effects of ion transport channel proteins or hormone regulation. As a newly identified alkalinity tolerant regulator, the physiological mechanism of alkalinity tolerant regulation of *OsAld-Y* is still unclear. In view of the fact that *OsAld-Y* synergistically affects the function of photosynthetic source tissues by regulating sugar metabolism and chloroplast development, we speculate that under alkaline stress, this gene may maintain the energy supply required for Na^+^/K^+^ selective transport in the underground part by reshaping the distribution pattern of carbon assimilation products in leaves, thereby prolonging the survival time of seedlings. Specifically, *OsAld-Y* may indirectly affect the root plasma membrane H^+^-ATPase-dependent Na^+^ efflux and K^+^ retention processes by regulating the output efficiency of glycolysis intermediates in chloroplasts, and finally establish a photosynthetic carbon metabolism-ion homeostasis cascade response pathway. In addition, the molecular mechanism of interaction between promoter elements and transcription factors and the protein factors interacting with *OsAld-Y* also need to be further studied. Whether hormones are involved in regulating the response to alkaline stress should be investigated by creating multiple types of transgenic material.

## Conclusion

In this study, we combined QTL-seq with a high-density map of 1002 F_2_ progeny from the sensitive cultivar TX144 and the tolerant LD19. By applying QTL-seq resequencing and a 4,326-SNP high-density linkage map, we identified *qATS6* as the major seedling alkalinity tolerance QTL, which explained 15.33% of the phenotypic variance, equally. The candidate genes of QTL were identified by the expression profile data of rice expression profile database and the expression level of genes under alkali stress. Compared with the wild type, under alkalinity stress, a large amount of Na^+^ and K^+^ in the roots of the loss-of-function *OsAld-Y* were transferred to the leaves, resulting in the death of *osald-y* leaves due to osmotic pressure imbalance after 12 days of alkalinity stress. This provides a key theory for the molecular breeding of alkali-tolerant genes and the study of alkalinity tolerance molecular mechanism of LD19.

## Data Availability

The datasets presented in this study can be found in online repositories. The names of the repository/repositories and accession number(s) can be found in the article/**Supplementary Material**.

## Supplementary material

Supplementary Figure 1 A linkage map covering a total genetic distance of 2252.63 cM and 4326 SNP markers in the rice genome with an average distance between bin markers of 2.03 cM. *qRKC1*, *qSNC1*, *qSKC1-1*, *qSKC1-2*, *qSDS3*, *qRKC4*, *qSDS6-1*, *qRKC6*, *qSNC6*, *qSKC6*, *qSDS6-2*, *qRNC6*, *qRKC9* and *qSKC9* were detected based on a threshold of LOD>3.
