# Supplementary figures and images for: OsAld-Y on qATS6 links to alkalinity tolerance at the seedling stage in Oryza sativa L. ssp. Japonica
Source: Front Plant Sci. 2026 Mar 10;17:1716444. doi: 10.3389/fpls.2026.1716444 (PMC13008629; doi:10.3389/fpls.2026.1716444)

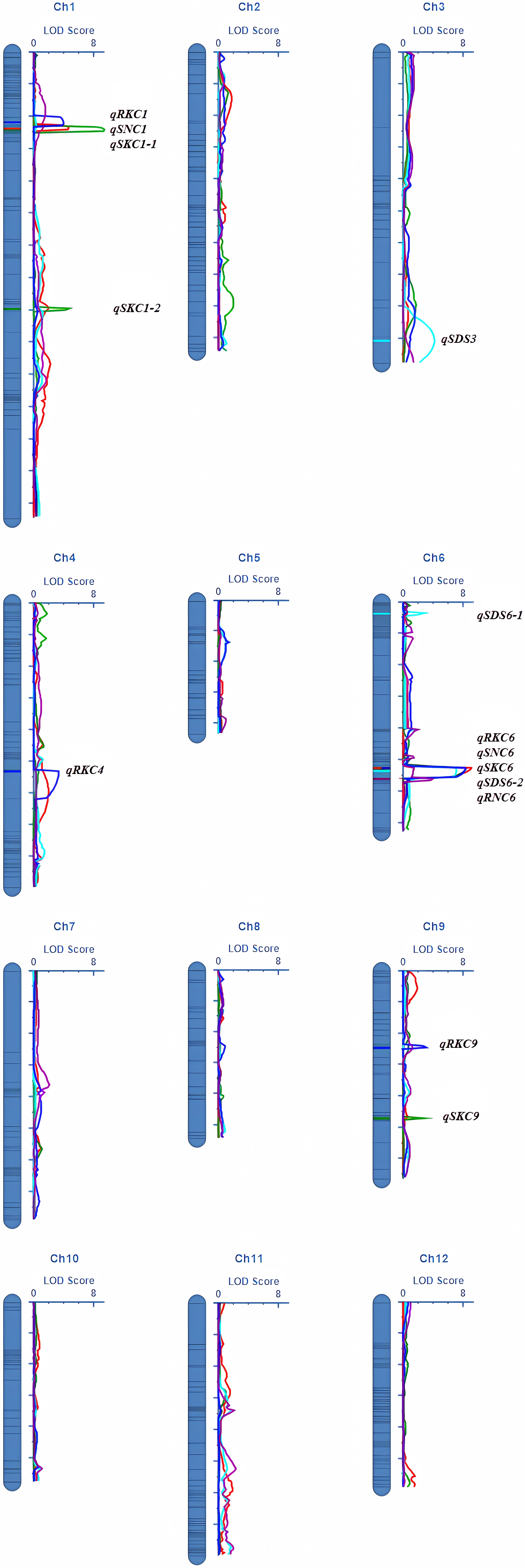

Supplement: Supplementary Figure 1 — A linkage map covering a total genetic distance of 2252.63 cM and 4326 SNP markers in the rice genome with an average distance between bin markers of 2.03 cM. qRKC1, qSNC1, qSKC1-1, qSKC1-2, qSDS3, qRKC4, qSDS6-1, qRKC6, qSNC6, qSKC6, qSDS6-2, qRNC6, qRKC9 and qSKC9 were detected based on a threshold of LOD>3. [file Image1.jpeg]
